# Hepato-protective effect of rutin via IL-6/STAT3 pathway in CCl_4_-induced hepatotoxicity in rats

**DOI:** 10.1186/s40659-015-0022-y

**Published:** 2015-06-11

**Authors:** Mohamed M. Hafez, Naif O. Al-Harbi, Ali Rashed Al-Hoshani, Khaled A. Al-hosaini, Shakir D. Al Shrari, Salim S. Al Rejaie, Mohamed M. Sayed-Ahmed, Othman A. Al-Shabanah

**Affiliations:** Department of Pharmacology and Toxicology, College of Pharmacy, King Saud University, P.O. Box 2457, Riyadh, 11451 Kingdom of Saudi Arabia

**Keywords:** Hepatotoxicity, Rutin, Inflammatory cytokine, MEK5, FADD, STAT3, JAK genes expression

## Abstract

**Background:**

Carbon tetrachloride (CCl_4_) induces hepatotoxicity in animal models, including the increased blood flow and cytokine accumulation that are characteristic of tissue inflammation. The present study investigates the hepato-protective effect of rutin on CCl_4_-induced hepatotoxicity in rats.

**Results:**

Forty male Wistar rats were divided into four groups. Group I (control group) received 1 mL/kg of dimethyl sulfoxide intragastrically and 3 mL/kg olive oil intraperitoneally twice a week for 4 weeks. Group II received 70 mg/kg rutin intragastrically. Groups III and IV received CCl_4_ (3 mL/kg, 30 % in olive oil) intraperitoneally twice a week for 4 weeks. Group IV received 70 mg/kg rutin intragastrically after 48 h of CCl_4_ treatment. Liver enzyme levels were determined in all studied groups. Expression of the following genes were monitored with real-time PCR: interleukin-6 (*IL-6*), dual-specificity protein kinase 5 (*MEK5*), Fas-associated death domain protein (*FADD*), epidermal growth factor (*EGF*), signal transducer and activator of transcription 3 (*STAT3*), Janus kinase (*JAK*), B-cell lymphoma 2 (*Bcl2*) and B-cell lymphoma-extra-large (*Bcl-XL*). The CCl_4_ groups showed significant increases in biochemical markers of hepatotoxicity and up-regulation of expression levels of *IL-6, Bcl-XL, MEK5, FADD, EGF, STAT3* and *JAK* compared with the control group. However, CCl_4_ administration resulted in significant down-regulation of *Bcl2* expression compared with the control group. Interestingly, rutin supplementation completely reversed the biochemical markers of hepatotoxicity and the gene expression alterations induced by CCl_4_.

**Conclusion:**

CCl_4_ administration causes alteration in expression of IL-6/STAT3 pathway genes, resulting in hepatotoxicity. Rutin protects against CCl_4_-induced hepatotoxicity by reversing these expression changes.

## Background

The liver plays an important role in regulating various physiological processes [1] and is involved in the detoxification of some drugs that may cause hepatotoxicity [2]. Carbon tetrachloride (CCl_4_) is a potent lipid-soluble hepatotoxic agent. Oxidative stress induced by CCl_4_ can cause cell damage and subsequent cell death, through oxidation of cellular components, such as lipids, proteins, and DNA [3]. CCl_4_ also produces peroxidative degeneration of many tissues when bound to lipids and proteins [4]. Exposure to CCl_4_ causes hepatocyte injury through metabolic activation of reactive oxygen species (ROS), such as superoxide anion, hydroxyl radicals, hydrogen peroxide, and other radicals generated during numerous metabolic reactions [5]. ROS are thought to be a major cause of this tissue damage. Oxidative stress resulting from increased free radical production after CCl_4_ administration may play an important role in the degenerative processes in the tissues [6]. It has been observed that the toxicity of CCl_4_ probably depends on the formation of the trichloromethyl radical (CCl_3_), which forms the more toxic CCl_3_O_2_ in the presence of oxygen [7]. ROS can induce tissue injury via lipid peroxidation, and enhance liver fibrosis by increasing tissue inhibitors of metalloproteinases (TIMP-1), which leads to an increase in collagen synthesis and accumulation [8, 9].

The acute and chronic phases of inflammation are characterized by specific humoral and cellular immune responses [10, 11]. The immune response is regulated by a complex network of cytokines and cytokine inhibitors [12]. Under normal conditions, cytokine inhibitors serve as immuno-modulators that limit the deleterious effects of excess inflammatory reactions [13]. Under pathologic conditions, anti-inflammatory mediators may provide insufficient control over pro-inflammatory activities or overcompensate and inhibit the immune response, rendering the host at risk from systemic infection [14]. Cytokine production can be stimulated by the activation of nuclear factor kappa β (NFκβ) and activator protein-1 (AP-1), which control physiological processes such as cell differentiation and proliferation [15]. Additionally, tumor necrosis factor alpha and interleukin-1 (IL-1) can lead to the activation of NFκβ expression [16] through phosphorylation of NFκβ signaling [17]. Cytokines provide hepato-protection in a variety of liver-injury models involving peroxidative degeneration of many tissues [18, 19]. Interleukin-6 (IL-6) is an inflammatory cytokine that regulates multiple biologic activities including the induction of acute-phase proteins in the liver [20]. In animal models of hepatotoxicity, IL-6 acts on hepatocytes to stimulate liver regeneration and repair. Secreted IL-6 binds to its receptor through the gp130 receptor, activating JAK. Activated JAK triggers the mitogen-activated protein kinases pathway, which is activated by SHP2–GRB2-SOS–Ras signal transduction, and triggers the STAT3 pathway, which is activated through JAK-mediated tyrosine phosphorylation. In this manner the STAT3 transcription factor dimerizes and translocates to the nucleus, where it activates the transcription of some target genes. In the liver, this process promotes liver regeneration, the acute-phase response and hepato-protection against Fas and toxic damage [21]. The activation of the caspase cascade, which results from the interaction of Fas with its receptor, is blocked by IL-6 and STAT3 through the up-regulation of pro-apoptotic genes [22].

The common mechanisms of hepato-protection target either Fas-mediated or toxin-mediated acute liver injury. Immune response-mediated liver damage occurs via the binding of specific ligands to their corresponding receptors, activating the Fas apoptotic pathway. Fas-associated protein with death domain is apoptotic and is implicated in innate immunity, inflammation, and tumor development [23]. B-cell lymphoma 2 (*Bcl-2*) gene family members are important regulators of apoptosis: intense inflammation induces pro-apoptotic proteins, with inhibition of anti-apoptotic *Bcl-2* [24].

CCl_4_ is a xenobiotic used to study hepatotoxicity in animal models by initiating lipid peroxidation and inflammation [25]. The bio-activation of the phase I cytochrome P450 system, induced by CCl_4_, may cause acute and chronic tissue injury through the formation of reactive metabolic trichloromethyl radicals. These radicals react with sulfhydryl groups (glutathione and protein thiols) and antioxidant enzymes. The over-production of trichloromethyl free radicals enhances membrane lipid peroxidation, ultimately leading to liver steatosis, fibrosis, or cirrhosis [26].

Flavonoids are found in fruits, vegetables, and medicinal plants and have an important role in the detoxification of free radicals [27, 28]. Rutin, a flavonoid glycoside, protects against CCl_4_-induced liver injuries in rats [25]. Khan et al. showed that the administration of two different doses of rutin, 50 and 70 mg/kg, after 48 h of treatment with 3mL/kg of 30 % CCl_4_ twice a week for 4 weeks, increased levels of endogenous liver antioxidant enzymes such as catalase superoxide dismutase, glutathione peroxidase, glutathione-S-transferase, glutathione reductase, and glutathione contents; and decreased lipid peroxidation [25]. Our recent study showed that CCl_4_ administration causes aberrations in the expression of genes involved in the oxidative stress pathway, resulting in DNA damage and hepatotoxicity. Rutin protects against this by enhancing antioxidant genes [29]. Furthermore, rutin has antitumor activity via its cytotoxic effects on SW480 cells; ameliorates the toxic effects of SW480 tumors in mice; and exerts anti-angiogenic properties [30]. The anti-inflammatory properties of rutin also counter the increased expression levels of inflammatory markers induced by a high-cholesterol diet [31]. The anti-mutagenic potential of rutin has been studied and it is suggested that rutin is chemo-preventive of phospholipase A2-mediated mutagenesis of heterocyclic amines [32].

The aim of the present study is to assess whether rutin prevents CCl_4_-induced hepatotoxicity in rats via the IL-6/STAT3 pathway, as well as to investigate the effectiveness of rutin against CCl_4_-hepatotoxicity.

## Methods

CCl_4_ and rutin were purchased from Sigma Chemicals (Sigma Aldrich Louis, MO, USA). The SYBR® Green PCR Master Mix kit was purchased from Applied Biosystems (Life Technologies, Grand Island, NY, USA) and primers used in this study were designed using Primer Express 3.0 software (Applied Biosystem, Life Technologies, Grand Island, NY, USA)) and synthesized by Metabion Company (Metabion international AG, semmelweisstrasse 3, planegg/steinkirchen Germany).

### Animals

Six-week-old male Wistar rats (average body weight 180–200 g) were obtained from the Animal Care Center, College of Pharmacy, King Saud University, Riyadh, Saudi Arabia. The animals were kept under standard conditions of temperature (22 ± 1 °C), humidity (50–55 %), and a 12-h light: dark cycle, with free access to standard laboratory feed and water, according to the study protocol. All methods were conducted in accordance with the *Guide for Care and Use of Laboratory Animals*, Institute for Laboratory Animal Research, National Institute of Health (NIH publication No. 80–23; 1996). The study was approved by the Research Ethics Committee of the College of Pharmacy (number 140/2014), King Saud University, Riyadh, Saudi Arabia.

### Experimental design

The experimental design follows Khan et al. [25]. Forty adult male Wistar rats were randomly divided into four groups of 10 animals each as follows.

Group I, the control group, received 3 mL/kg olive oil (intraperitoneally; Monday and Thursday) and 3 mL/kg DMSO (intragastrically using gavage) twice a week for 4 weeks (Saturday and Wednesday).

Group II, the rutin group, was intragastrically treated with 70 mg/kg rutin in DMSO twice a week for 4 weeks (Saturday and Wednesday).

Group III, the CCl_4_ group, was intraperitoneally treated with 3 mL/kg CCl_4_ (30 % in olive oil) twice a week (Monday and Thursday) for 4 weeks.

Group IV, the CCl_4_-rutin group, intragastrically received 70 mg/kg rutin, after 48 h of CCl_4_ treatment, twice a week (Saturday and Wednesday) for 4 weeks.

At least 24 h after the last treatment protocol, all animals were exposed to ether and killed by decapitation. The blood samples were obtained and the sera were separated and kept at −80 °C until used for the bioassays. The liver was immediately removed then washed with ice-cold saline solution. Part of the liver was snap frozen in liquid nitrogen and stored until used for the gene expression analysis.

### Bioassays

The serum levels of liver enzymes (aspartate aminotransferase (AST), alanine aminotransferase (ALT)) were estimated using commercially available diagnostic kits (Human, Wiesbaden, Germany).

### Detection of gene expression in liver tissue with real-time PCR

#### Total RNA extraction

Total RNA was extracted from liver tissues using TRIzol method according to the manufacturer’s protocol. In brief, RNA was extracted by homogenization (Omni, Omni International, USA) in TRIzol reagent (Invitrogen, Life Technologies, USA) at maximum speed for 90–120 s. The homogenate was then incubated for 5 min at room temperature. A 1:5 volume of chloroform was added, and the tube was vortexed and centrifuged at 12 000 g for 15 min. The aqueous phase was isolated, and the total RNA was precipitated with absolute ethanol. After centrifugation and washing, the total RNA was finally eluted in 20 μL of the RNase-free water. The RNA concentrations and purity were measured with an ultraviolet spectrophotometer (NanoDrop 8000, Thermo Scientific, USA). The extracted RNA had a 260:280 ratio of 1.9–2.1.

#### Complementary DNA synthesis and real-time quantitative PCR

The cDNA was synthesized from 1 μg RNA using SuperScript III First-Strand Synthesis System as described in the manufacturer’s protocol (Invitrogen, Life Technologies). In brief, 1 μg of total RNA was mixed with 50 μM oligo (dT)_20_, 50 ng/μL random primers, and 10 mM dNTP mix in a total volume of 10 μL. The mixture was incubated at 56 °C for 5 min, then placed on ice for 3 min. The reverse transcriptase master mix containing 2 μL of 10× RT buffer, 4 μL of 25 mM MgCl_2_, 2 μL of 0.1 M DTT, and 1 μL of SuperScript® III RT (200 U/μL) was added to the mixture and was incubated at 25 °C for 10 min followed by 50 min at 50 °C.

Real-time quantitative PCR (SYBR® Green PCR Master Mix kit) was used to detect the expression levels of *IL-6, MEK5, FADD, EGF, STAT3, JAK, Bcl2*, and *Bcl-XL* genes in the liver tissue. The reaction was performed on an ABI 7500 Detection System (Applied Biosystems, Life Technologies, Grand Island, NY, USA)). The program was set to run for one cycle at 95 °C for 2 min, followed by 40 cycles at 95 °C for 15 s and 60 °C for 1 min. The specificity of the PCR amplification was confirmed by melting curve analysis. *GAPDH* was used as an internal control for qRT-PCR. The primers used in this study are listed in Table 1. The results of gene expression were analyzed using the 2^-ΔΔCT^ method [29]. The data were expressed as mean fold changes ± standard error for three independent amplifications.

**Table 1:** Primers used in real-time PCR

Gene name	Forward primer	Reverse primer
*IL-6*	5′-ATCTGCCCTTCAGGAACAGC-3′	5′-AGCCTCCGACTTGTGAAGTG-3′
*STAT3*	5′-CAAAGAAAACATGGCCGGCA-3′	5′-GGGGGCTTTGTGCTTAGGAT-3′
*JAK-1*	5′-ATGGAGTTTCTGCCTTCGGG-3′	5′-TTCTTGCTGCTAAGTCCCGG-3′
*MEK5*	5′-TCGTGCCATGGAGAACCA-3′	5′-CGCGCCACTATTTGGAATCT-3′
*FADD*	5′-CCAAACAAGTGCAAGAGCCC-3′	5′-AGGATTGCAGAGTGAGCCAC-3′
*Bcl2*	5′-CTTCTCTCGTCGCTACCGTC-3′	5′-CATGACCCCACCGAACTCAA-3′
*BCL-XL*	5′-CTTCTCTCGTCGCTACCGTC-3′	5′-GTGAGGTGGAAGGGACCATG-3′
*EGF*	5′-TGTGGGCTGAGAAGAAGCTG-3′	5′-GAGTACCAGATCTGCCGCTC-3′
*GAPDH*	5′-AACTCCCATTCCTCCACCTT-3′	5′-GAGGGCCTCTCTCTTGCTCT-3′

### Statistical analyses

The differences between the obtained values (mean ± SEM, n = 10) was assessed with one-way analysis of variance followed by the Tukey–Kramer multiple comparison using Graph pad prism 5 software (GraphPad Software, Inc., La Jolla, CA, USA) The differences were considered statistically significant when *p* < 0.05.

## Results

**Fig. 1:**
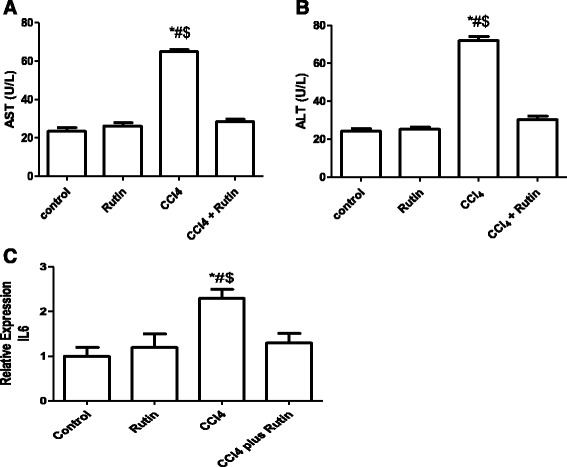
The Effect of CCl_4_, rutin, and their combination on the serum levels of AST (**a**), ALT (**b**) and expression levels of IL6 in rat liver (**c**). Data were presented as mean ± SEM (n = 10). *, # and $ indicate significant change from control, rutin and CCl_4_ plus rutin, respectively, at *P* < 0.05 using ANOVA followed by Tukey–Kramer as a post ANOVA test

The liver enzymes (ALT and AST levels) in sera were used as biochemical markers for early acute hepatotoxicity. The CCl_4_ group showed a significant increase in the levels of AST (65 ± 1.2 U/L) Fig. 1a and ALT (72 ± 2.2 U/L) Fig. 1b compared with the control group (23.5 ± 1.8 U/L and 24.2 ± 1.3 U/L, respectively) (*p* < 0.001). However, rutin restored levels of biomarkers of CCl_4_-induced hepatic damage to their normal values, as indicated by the control group.

The effect of CCl_4_, rutin, and their combination on *IL-6* gene expression in liver tissues using RT-PCR is illustrated in Fig. 1c. *IL-6* expression in the CCl_4_ group showed a significant increase of 130 % compared with the control group (*p* < 0.01). The administration of rutin alone resulted in a non-significant increase in the *IL-6* level compared with the control group. Interestingly, the CCl_4_-rutin group showed complete reversal of the CCl_4_-induced increase and showed a significant decrease in *IL-6* gene expression level of 44 % (*p* < 0.02) compared with CCl_4_.

**Fig. 2:**
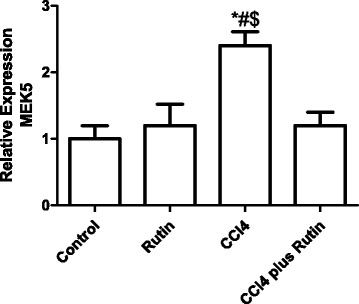
The Effect of CCl4, rutin, and their combination on the expression levels of MEK5 gene in rat liver. Data were presented as mean ± SEM (*n* = 10). *, # and $ indicate significant change from control, rutin and CCl4 plus rutin, respectively, at *P* < 0.05 using ANOVA followed by Tukey–Kramer as a post ANOVA test

The effect of CCl_4_, rutin, and their combination on *MEK5* gene expression is shown in Fig. 2. Expression of *MEK5* in the CCl_4_ group increased significantly by 140 % (*p* < 0.002) compared with the control group. The CCl_4_-rutin group showed a significant decrease in *MEK5* expression of 50 % (*p* < 0.001) compared with the CCl_4_ group. This change in *MEK5* was not significant (*p* < 0.8) compared with the control group.

**Fig. 3:**
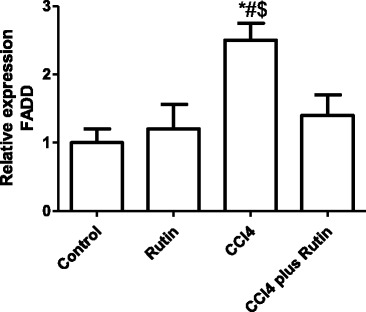
The Effect of CCl4, rutin, and their combination on the expression levels of FADD gene in rat liver. Data were presented as mean ± SEM (*n* = 10). *, # and $ indicate significant change from control, rutin and CCl4 plus rutin, respectively, at *P* < 0.05 using ANOVA followed by Tukey–Kramer as a post ANOVA test

The effect of CCl_4_, rutin, and their combination on *FADD* gene expression is illustrated in Fig. 3. The CCl_4_ group showed an increase in *FADD* expression of 150 % (*p* < 0.0002) compared with the control group, and of 108 % (*p* < 0.007) compared with the rutin group. However, the rutin-CCl4 group showed a complete reversal of the *FADD* expression increase. This reversal resulted in a significant decrease in *FADD* expression by 40 % (*p* < 0.007) compared with the CCl_4_ group.

**Fig. 4:**
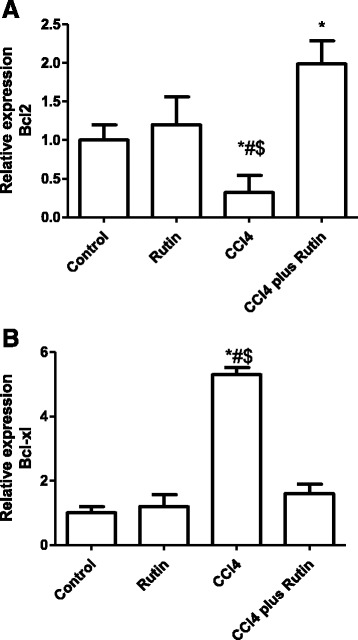
The Effect of CCl4, rutin, and their combination on the expression levels of Bcl2 (**a**) and Bcl-xl (**b**) genes in rat liver. Data were presented as mean ± SEM (*n* = 10). *, # and $ indicate significant change from control, rutin and CCl4 plus rutin, respectively, at *P* < 0.05 using ANOVA followed by Tukey–Kramer as a post ANOVA test

The effect of CCl_4_, rutin, and their combination on *Bcl2* and *Bcl-XL* expression is shown in Fig. 4 (a & b). In the CCl_4_ group, *Bcl2* expression significantly decreased (68 %; *p* < 0.03) and *Bcl-XL* expression level increased (430 %; *p* < 0.001) compared with the control group. The CCl_4_-rutin group showed complete reversal of the *Bcl-XL* increase and a significant increase in *Bcl2* expression levels of 99 % compared with the control group. This reversal involved a significant increase in *Bcl2* expression of 522 % (*p* < 0.003) and a significant decrease in *Bcl-XL* expression of 70 % (*p* < 0.0001) compared with the CCl_4_ group.

**Fig. 5:**
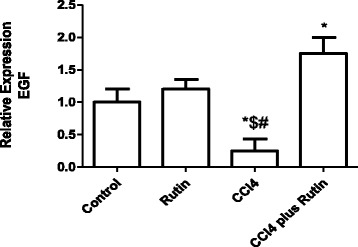
The Effect of CCl4, rutin, and their combination on the expression levels of EGF gene in rat liver. Data were presented as mean ± SEM (*n* = 10). *, # and $ indicate significant change from control, rutin and CCl4 plus rutin, respectively, at *P* < 0.05 using ANOVA followed by Tukey–Kramer as a post ANOVA test

The effect of CCl_4_, rutin, and their combination on *EGF* expression level is shown in Fig. 5. *EGF* expression decreased significantly in the CCl_4_ group compared with the control group (75 %; *p* < 0.02) and the rutin group (79 %; *p* < 0.005). In the CCl_4_-rutin group, EGF expression was completely restored to its normal levels, observed as a significant increase in *EGF* expression of 600 % (*p* < 0.0003) compared with the CCl_4_ group, restoring it to 75 % (*p* < 0.03) of control group levels.

**Fig. 6:**
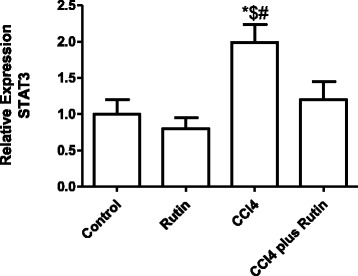
The Effect of CCl4, rutin, and their combination on the expression levels of STAT3 gene in rat liver. Data were presented as mean ± SEM (*n* = 10). *, # and $ indicate significant change from control, rutin and CCl4 plus rutin, respectively, at *P* < 0.05 using ANOVA followed by Tukey–Kramer as a post ANOVA test

Figure 6 shows the effect of CCl_4_, rutin, and their combination on *STAT3* expression. In the CCl_4_ group, there was a significant increase in *STAT3* expression by 99 %, compared with the control group (*p* < 0.006). Whereas administration of rutin in combination with CCl_4_ significantly decreased the *STAT3* expression to 40 % of the CCl_4_ group levels (*p* < 0.04).

**Fig. 7:**
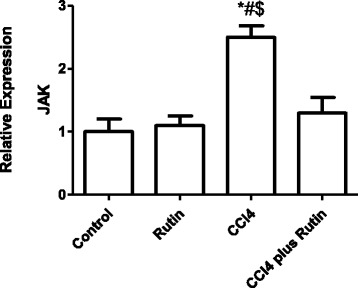
The Effect of CCl4, rutin, and their combination on the expression levels of JAK gene in rat liver. Data were presented as mean ± SEM (*n* = 10). *, # and $ indicate significant change from control, rutin and CCl4 plus rutin, respectively, at *P* < 0.05 using ANOVA followed by Tukey–Kramer as a post ANOVA test

Figure 7 shows the effect of CCl_4_, rutin, and their combination on *JAK* gene expression in liver tissues. In the CCl_4_ group, there was a significant increase the *JAK* expression of 150 % (*p* < 0.005) compared with the control group. The supplementation of rutin in combination with CCl_4_ resulted in complete reversal of the CCl_4_ effect: *JAK* expression returned to its normal values. This reversal was observed as a significant decrease in *JAK* expression of 48 % (*p* < 0.05) compared with the CCl_4_ group. In the rutin group, an insignificant increase in *JAK* expression compared with the control group was observed.

## Discussion

The liver is the first line of protection against damage, which may lead to hepatic necrosis and apoptosis, induced by xenobiotics and drugs [33]. The release of hepatocellular leakage enzymes is used as a marker for hepatotoxicity. CCl_4_ is widely used to investigate the liver injury that is associated with oxidative stress and free radicals. The reactive oxygen species induced by CCl_4_ not only cause direct tissue damage, but also initiate inflammation through the activation of various cytokines [34].

Several studies have focused on the prevention of CCl_4_-induced hepatotoxicity [35–37]. The current study showed significant increases in the serum levels of ALT and AST as a result of CCl_4_ administration. This agrees with previous studies that demonstrated significant increases in ALT and AST levels in CCl_4_-treated rats and mice compared with untreated ones [38–40]. This increase in liver enzymes may be owing to acute hepatocyte injury caused by CCl_4_ [41]. Rutin has been shown to have hepato-protective activity, possibly protecting the liver from CCl_4_-induced injury, as rutin given after CCl_4_ significantly restores the elevated AST and ALT levels. Similarly, previous studies have reported the protective effect of flavonoid compounds against a high-cholesterol diet and CCl_4_-induced hepatotoxicity [25, 42].

Immune-mediated liver damage occurs via the activation of the Fas apoptotic death pathway. The link between Fas-mediated damage and the induction of ROS with oxidative stress has been established [43]. The apoptotic pathway is initiated when specific ligands bind to their corresponding receptors. The FADD is an adaptor transmitting apoptotic signals mediated by the death receptors (DR), which lead to two cell death pathways [23]. The death-inducing signaling complex is composed of FADD and procaspase-8, and facilitates the activation of both procaspase-8 and −10 [44]. Their activation leads to proteolytic stimulation of caspase-3, −6, and −7, which can cleave intracellular substrates [44, 45]. In the present study, *FADD* was significantly increased in the CCl_4_ group. Similarly, Jiang et al. found that the administration of 1 mL/kg of CCl_4_ in olive oil twice a week for 4 weeks led to a significant increase in the expression of *FADD* compared with the olive oil-only control group [46]. Similarly, CCl_4_ increases expression of *Fas/FasL* and increases the activities of caspase-3 and-8 and cytochrome P4502E1, which leads to liver apoptosis [47]. Fas binds to its ligand and forms the death-inducing signaling complex via FADD and then activates caspase-8, which leads to activation of caspase-9 and −3 [48]. The reduced *FADD* gene expression as a result of rutin supplementation indicates that rutin decreases the CCl_4_-induced hepatocellular damage that might be through its anti-apoptotic effect.

The Bcl2 family members are important regulators for apoptosis and inflammation [24]. In CCl_4_-induced hepatotoxicity, genes for JNK play an essential role in modulating the pro- and anti-apoptotic proteins located in the mitochondria. JNK, together with ROS, can stimulate pro-apoptotic proteins and can promote apoptosis by inhibiting anti-apoptotic proteins [49, 50]. In the current study, the suppression of *Bcl2* expression, as a result of CCl_4_, leads to hepatotoxicity and apoptosis. The increased *Bcl2* gene expression in liver tissue after rutin supplementation in CCl_4_-treated rats suggests that rutin may protect against CCl_4_-induced hepatotoxicity by regulating JNK signaling and mitochondrial intrinsic apoptotic pathways. A similar study has found that CCl_4_ reduced *Bcl2* expression in association with increased *Bax* expression and *Bax/Bcl2* ratio [51]. Furthermore, rutin can cause tumor cell apoptosis through a decrease in *Bcl2* expression and the *Bax/Bcl2* ratio [52]. These results suggest that the key role of rutin in inducing the apoptosis of tumor cells is through the regulation of the Bcl2/BAX balance.

Cytokine production in the liver depends on the initial induction of early-response cytokines [53]. IL-6 helps hepatic survival by stimulating liver recovery and gives hepato-protection [18, 19]. The binding of IL-6 to its receptor (IL-6R) prompts STAT3 pathway activation through binding to glycoprotein 130 (gp130). Another alternative pathway is via the IL-6 signal (IL-6 trans-signaling) [54]. IL-6 acts as both a pro- and an anti-inflammatory cytokine and may mediate liver damage through different pathways. In the current study, CCl_4_ significantly upregulated IL-6 expression, whereas rutin administration suppressed this change, which may be because of its anti-inflammatory activity. A similar study in rats showed that curcumin supplementation suppressed CCl_4_-induced IL-6 production by the prevention of pro-inflammatory cytokine secretion [55]. Elevated levels of IL-6 are associated with disease states [40]. The soluble form of IL-6R, in addition to the membrane-bound receptor, binds to IL-6 and prolongs its plasma half-life [56]. The soluble IL-6R has roles in cellular proliferation, differentiation, and activation of inflammatory responses [57, 58]. Signal transducer and activator of transcription-3 mediates signal transduction and is regulated by IL-6 [21]. The IL-6/IL-6R complex promotes the initiation of STAT3 by JAK, resulting in DNA binding of STAT3 [59, 60].

STAT3 and its upstream JAK signaling mediates the immune responses of various cytokines and participates in inflammation, cell growth, and metastasis [61]. IL-6/STAT3 can be activated by other cytokines such as IL-11 [62]. The fully activated STAT3 regulates gene transcription of anti-apoptotic (Bcl-XL) [63, 64] and proliferation (cyclin D1 and Myc) regulatory proteins [65, 66]. In the current study, CCl_4_ increased the expression of *STAT3*, which led to *Bcl2* down-regulation and *Bcl-XL* up-regulation. Similarly, another study reported that the activation of *STAT3* increases *Bcl2* mRNA and protein expression [63]. The activation of *Bcl-X* produces two different Bcl-XL proteins. The longer form Bcl-X (Bcl-XL) becomes a repressor of apoptosis, whereas the shorter form (Bcl-XS) can enhance apoptosis [67, 68]. Administration of rutin in combination with CCl_4_ resulted in suppression of *STAT3* and over-expression of *Bcl2,* which reduces the apoptosis. Similarly, rutin inhibits inflammatory responses in ultraviolet-irradiated mouse skin by inhibiting the levels of phosphorylated STAT3 [69]. This indicates that rutin may play an important role in protection against CCl_4_-induced hepatotoxicity.

Many inflammatory cytokines play important roles in regulating liver fibrogenesis [70]. IL-6, interferon-γ (IFN-γ), IFN-α/β, and IL-22 are involved in MEK5-ERK5 and JAK-STAT3 pathway activations [71, 72]. The increased expression of *MEK5* and *JAK* in the CCl_4_-treated group, and the restoration to control levels after treatment with rutin, suggests that genes related to both the JAK-STAT3 and the MEK5-ERK5 pathways were overexpressed as a result of IL-6 expression in response to CCl_4_-induced hepatotoxicity. A similar study has shown CCl_4_-induced IL-6 activation is associated with an increase in *MEK5, ERK5, JAK*, and *STAT3* expression prior to cirrhosis. These alterations can be reversed by silymarin treatment, thus lowering liver cirrhosis [73]. Several studies have revealed that the activation of the JAK-STAT pathway by cytokines has been shown to regulate fibrogenic cytokines such as transforming growth factor-β1 and connective tissue growth factor [74], as well as enhancing liver fibrosis and cancer [75–77].

Epidermal growth factor and its tyrosine kinase receptor (EGFR) are proposed to have essential roles in liver regeneration and transformation [78, 79]. EGF and EGFR are highly elevated in human cirrhotic livers [80]. Activated EGF, with other cytokines, stimulates the production of TIMP-1 [81]. *TIMP-1* is expressed during liver injury by the activated hepaticstellate cells and Kupffer cells that are the major sources of TIMP-1 [82]. In the present study, CCl_4_ increases *TIMP-1* expression so *EGF* expression decreases, leading to liver fibrosis due to the accumulation of collagen in the liver. Similarly, in mice, the mRNA and protein expression of *EGF* were significantly decreased during liver injury by CCl_4_ but increased during repair [83]. Another study showed a significant increase in *EGF* expression in rats during the course of cirrhosis development [84]. The expression levels of *TIMP-1* in hepatocytes during CCl_4_-induced hepatotoxicity are controlled by STAT3. A similar study has suggested that STAT3 activation in hepatocytes plays an important role in induction of *TIMP-1* during liver injury [85]. Therefore, rutin administration enhanced collagen-lysis activity as a result of the decrease in EGF expression and the corresponding decrease in *TIMP-1* expression. In another study, rutinwas directly bounded with EGFR and down-regulated its protein levels [86].

## Conclusion

This study demonstrates that rutin has potent protective effects against CCl_4_-induced hepatotoxicity by restoring the alteration in expression of genes in the IL-6/STAT3 pathway through its anti-apoptotic, anti-inflammatory and anti-oxidant effects. This study suggests that rutin may be used as an alternative treatment for liver diseases.

## References

[1] Thilakchand KR, Mathai RT, Simon P, Ravi RT, Baliga-Rao MP, Baliga MS (2013). Hepatoprotective properties of the Indian gooseberry (Emblica officinalis Gaertn): a review. Food Funct.

[2] Kamisan FH, Yahya F, Mamat SS, Kamarolzaman MF, Mohtarrudin N, Kek TL (2014). Effect of methanol extract of Dicranopteris linearis against carbon tetrachloride- induced acute liver injury in rats. BMC Complement Altern Med.

[3] Hsu DZ, Li YH, Chu PY, Chien SP, Chuang YC, Liu MY (2006). Attenuation of endotoxin-induced oxidative stress and multiple organ injury by 3,4-Methylenedioxyphenol in rats. Shock.

[4] Ritter C, Reinke A, Andrades M, Martins MR, Rocha J, Menna-Barreto S (2004). Protective effect of N-acetylcysteine and deferoxamine on carbon tetrachloride-induced acute hepatic failure in rats. Crit Care Med.

[5] Kohen R, Nyska A (2002). Oxidation of biological systems: oxidative stress phenomena, antioxidants, redox reactions, and methods for their quantification. Toxicol Pathol.

[6] Al-Rasheed NM, Faddah LM, Mohamed AM, Mohammad RA, Al-Amin M (2014). Potential impact of silymarin in combination with chlorogenic acid and/or melatonin in combating cardiomyopathy induced by carbon tetrachloride. Saudi J Biol Sci.

[7] Ganie SA, Haq E, Hamid A, Qurishi Y, Mahmood Z, Zargar BA (2011). Carbon tetrachloride induced kidney and lung tissue damages and antioxidant activities of the aqueous rhizome extract of Podophyllum hexandrum. BMC Complement Altern Med.

[8] Adesanoye OA, Farombi EO (2010). Hepatoprotective effects of Vernonia amygdalina (astereaceae) in rats treated with carbon tetrachloride. Exp Toxicol Pathol.

[9] Blomhoff R (2005). Dietary antioxidants and cardiovascular disease. Curr Opin Lipidol.

[10] Watelet JB, El Shazly A, Collet S, Doyen A (2012). Chronic inflammation of upper airways in children: basic principles. B-ENT.

[11] Wallace GR, Niemczyk E (2011). Genetics in ocular inflammation–basic principles. Ocul Immunol Inflamm.

[12] Allampallam K, Shetty V, Mundle S, Dutt D, Kravitz H, Reddy PL (2002). Biological significance of proliferation, apoptosis, cytokines, and monocyte/macrophage cells in bone marrow biopsies of 145 patients with myelodysplastic syndrome. Int J Hematol.

[13] Johnston GR, Webster NR (2009). Cytokines and the immunomodulatory function of the vagus nerve. Br J Anaesth.

[14] Munoz C, Carlet J, Fitting C, Misset B, Bleriot JP, Cavaillon JM (1991). Dysregulation of in vitro cytokine production by monocytes during sepsis. J Clin Invest.

[15] Elmarakby AA, Sullivan JC (2012). Relationship between oxidative stress and inflammatory cytokines in diabetic nephropathy. Cardiovasc Ther.

[16] Valacchi G, Pagnin E, Phung A, Nardini M, Schock BC, Cross CE (2005). Inhibition of NFkappaB activation and IL-8 expression in human bronchial epithelial cells by acrolein. Antioxid Redox Signal.

[17] Tapalaga D, Tiegs G, Angermuller S (2002). NFkappaB and caspase-3 activity in apoptotic hepatocytes of galactosamine-sensitized mice treated with TNFalpha. J Histochem Cytochem.

[18] Jiang MD, Ma HD, Zhong XF, Xie FW, Zeng WZ (2003). [Effects of Erk signal transduction on the cell cycle of rat hepatic stellate cells stimulated by acetaldehyde]. Zhonghua gan zang bing za zhi = Zhonghua ganzangbing zazhi =Chinese journal of hepatology.

[19] Saile B, Eisenbach C, El-Armouche H, Neubauer K, Ramadori G (2003). Antiapoptotic effect of interferon-alpha on hepatic stellate cells (HSC): a novel pathway of IFN-alpha signal transduction via Janus kinase 2 (JAK2) and caspase-8. Eur J Cell Biol.

[20] Platonov OM, Korotkoruchko VP, Polishchuk AS, Pinchuk VG (1970). [Transfer of nuclear DNA-like RNA into the cytoplasm in process of liver regeneration and chemically induced liver neoplasm]. Ukr Biokhim Zh.

[21] Taub R (2003). Hepatoprotection via the IL-6/Stat3 pathway. J Clin Invest.

[22] Chen Q, Ray S, Hussein MA, Srkalovic G, Almasan A (2003). Role of Apo2L/TRAIL and Bcl-2-family proteins in apoptosis of multiple myeloma. Leuk Lymphoma.

[23] Tourneur L, Chiocchia G (2010). FADD: a regulator of life and death. Trends Immunol.

[24] Tsujimoto Y, Shimizu S (2000). Bcl-2 family: life-or-death switch. FEBS Lett.

[25] Khan RA, Khan MR, Sahreen S (2012). CCl4-induced hepatotoxicity: protective effect of rutin on p53, CYP2E1 and the antioxidative status in rat. BMC Complement Altern Med.

[26] Hefnawy TM, Mohammed FR (2013). Protective effects of Lactuca sativa ethanolic extract on carbon tetrachloride induced oxidative damage in rats. Asian Pac J Trop Dis.

[27] Potter JD (1997). Cancer prevention: epidemiology and experiment. Cancer Lett.

[28] Huntley AL (2009). The health benefits of berry flavonoids for menopausal women: cardiovascular disease, cancer and cognition. Maturitas.

[29] Hafez MM, Al-Shabanah OA, Al-Harbi NO, Al-Harbi MM, Al-Rejaie SS, Alsurayea SM (2014). Association between paraoxonases gene expression and oxidative stress in hepatotoxicity induced by CCl4. Oxid Med Cell Longev.

[30] Alonso-Castro AJ, Dominguez F, Garcia-Carranca A (2013). Rutin exerts antitumor effects on nude mice bearing SW480 tumor. Arch Med Res.

[31] Sikder K, Kesh SB, Das N, Manna K, Dey S (2014). The high antioxidative power of quercetin (aglycone flavonoid) and its glycone (rutin) avert high cholesterol diet induced hepatotoxicity and inflammation in Swiss albino mice. Food Funct.

[32] Bear WL, Teel RW (2000). Effects of citrus flavonoids on the mutagenicity of heterocyclic amines and on cytochrome P450 1A2 activity. Anticancer Res.

[33] Tacke F, Luedde T, Trautwein C (2009). Inflammatory pathways in liver homeostasis and liver injury. Clin Rev Allergy Immunol.

[34] Zhang JM, An J (2007). Cytokines, inflammation, and pain. Int Anesthesiol Clin.

[35] Aruoma OI (2003). Methodological considerations for characterizing potential antioxidant actions of bioactive components in plant foods. Mutat Res.

[36] Emzhik M, Rahimi-Moghaddam P, Ebrahimi SA, Keyhanfar F, Moazzam AS (2015). Commentary on prevention a possible drug-drug interaction: is concurrent administration of orlistat and pioglitazone increase the risk of durg-induced hepatotoxicity?. Int J Prevent Med.

[37] Ikeda T (2015). Idiosyncratic drug hepatotoxicity: strategy for prevention and proposed mechanism. Curr Med Chem.

[38] El-Sayed YS, Lebda MA, Hassinin M, Neoman SA (2015). Chicory (Cichorium intybus L.) Root Extract Regulates the Oxidative Status and Antioxidant Gene Transcripts in CCl4-Induced Hepatotoxicity. PLoS One.

[39] Na JY, Kim S, Song K, Kwon J (2015). Hepatoprotective effect of phosphatidylcholine against carbon tetrachloride liver damage in mice. Biochem Biophys Res Commun.

[40] Ganaie MA, Khan TH, Siddiqui NA, Ansari MN. Ameliorative effect of methanol extract of Rumex vesicarius on CCl-induced liver damage in Wistar albino rats. Pharm Biol. 2015;1–5. 10.3109/13880209.2014.967782 25702903

[41] Hu YY, Liu CH, Wang RP, Liu C, Liu P, Zhu DY (2000). Protective actions of salvianolic acid A on hepatocyte injured by peroxidation in vitro. World J Gastroenterol.

[42] Al-Rejaie SS, Aleisa AM, Sayed-Ahmed MM, Al-Shabanah OA, Abuohashish HM, Ahmed MM (2013). Protective effect of rutin on the antioxidant genes expression in hypercholestrolemic male Westar rat. BMC Complement Altern Med.

[43] Matsumura H, Shimizu Y, Ohsawa Y, Kawahara A, Uchiyama Y, Nagata S (2000). Necrotic death pathway in Fas receptor signaling. J Cell Biol.

[44] Slee EA, Adrain C, Martin SJ (2001). Executioner caspase-3, −6, and −7 perform distinct, non-redundant roles during the demolition phase of apoptosis. J Biol Chem.

[45] Widmann C, Gerwins P, Johnson NL, Jarpe MB, Johnson GL (1998). MEK kinase 1, a substrate for DEVD-directed caspases, is involved in genotoxin-induced apoptosis. Mol Cell Biol.

[46] Jiang Y, Liu J, Waalkes M, Kang YJ (2004). Changes in the gene expression associated with carbon tetrachloride-induced liver fibrosis persist after cessation of dosing in mice. J Soc Toxicol.

[47] Lin X, Huang R, Zhang S, Zheng L, Wei L, He M (2012). Methyl helicterate protects against CCl4-induced liver injury in rats by inhibiting oxidative stress, NF-kappaB activation, Fas/FasL pathway and cytochrome P4502E1 level. Food Chem Toxicol.

[48] Li G, Han C, Xu L, Lim K, Isse K, Wu T (2009). Cyclooxygenase-2 prevents fas-induced liver injury through up-regulation of epidermal growth factor receptor. Hepatology.

[49] Sinha K, Das J, Pal PB, Sil PC (2013). Oxidative stress: the mitochondria-dependent and mitochondria-independent pathways of apoptosis. Arch Toxicol.

[50] Ma JQ, Ding J, Zhang L, Liu CM (2014). Hepatoprotective properties of sesamin against CCl4 induced oxidative stress-mediated apoptosis in mice via JNK pathway. Food Chem Toxicol.

[51] Guo XL, Liang B, Wang XW, Fan FG, Jin J, Lan R (2013). Glycyrrhizic acid attenuates CCl(4)-induced hepatocyte apoptosis in rats via a p53-mediated pathway. World J Gastroenterol.

[52] Chen H, Miao Q, Geng M, Liu J, Hu Y, Tian L (2013). Anti-tumor effect of rutin on human neuroblastoma cell lines through inducing G2/M cell cycle arrest and promoting apoptosis. ScientificWorld Journal.

[53] Ramadori G, Armbrust T (2001). Cytokines in the liver. Eur J Gastroenterol Hepatol.

[54] Wolf, J., Rose-John, S. & Garbers, C. Interleukin-6 and its receptors: A highly regulated and dynamic system. *Cytokine* 2014. 10.1016/j.cyto.2014.05.024 24986424

[55] Reyes-Gordillo K, Segovia J, Shibayama M, Vergara P, Moreno MG, Muriel P (2007). Curcumin protects against acute liver damage in the rat by inhibiting NF-kappaB, proinflammatory cytokines production and oxidative stress. Biochim Biophys Acta.

[56] Peters M, Meyer zum Buschenfelde KH, Rose-John S (1996). The function of the soluble IL-6 receptor in vivo. Immunol Lett.

[57] Novick D, Engelmann H, Wallach D, Rubinstein M (1989). Soluble cytokine receptors are present in normal human urine. J Exp Med.

[58] Honda M, Yamamoto S, Cheng M, Yasukawa K, Suzuki H, Saito T (1992). Human soluble IL-6 receptor: its detection and enhanced release by HIV infection. J Immunol.

[59] Poli G, Bressler P, Kinter A, Duh E, Timmer WC, Rabson A (1990). Interleukin 6 induces human immunodeficiency virus expression in infected monocytic cells alone and in synergy with tumor necrosis factor alpha by transcriptional and post-transcriptional mechanisms. J Exp Med.

[60] Turner M, Chantry D, Feldmann M (1990). Transforming growth factor beta induces the production of interleukin 6 by human peripheral blood mononuclear cells. Cytokine.

[61] Darnell JE (1997). STATs and gene regulation. Science.

[62] Hirano T, Ishihara K, Hibi M (2000). Roles of STAT3 in mediating the cell growth, differentiation and survival signals relayed through the IL-6 family of cytokine receptors. Oncogene.

[63] Epling-Burnette PK, Liu JH, Catlett-Falcone R, Turkson J, Oshiro M, Kothapalli R (2001). Inhibition of STAT3 signaling leads to apoptosis of leukemic large granular lymphocytes and decreased Mcl-1 expression. J Clin Invest.

[64] Catlett-Falcone R, Landowski TH, Oshiro MM, Turkson J, Levitzki A, Savino R (1999). Constitutive activation of Stat3 signaling confers resistance to apoptosis in human U266 myeloma cells. Immunity.

[65] Niu G, Bowman T, Huang M, Shivers S, Reintgen D, Daud A (2002). Roles of activated Src and Stat3 signaling in melanoma tumor cell growth. Oncogene.

[66] Bowman T, Broome MA, Sinibaldi D, Wharton W, Pledger WJ, Sedivy JM (2001). Stat3-mediated Myc expression is required for Src transformation and PDGF-induced mitogenesis. Proc Natl Acad Sci U S A.

[67] Boise LH, Gonzalez-Garcia M, Postema CE, Ding L, Lindsten T, Turka LA (1993). bcl-x, a bcl-2-related gene that functions as a dominant regulator of apoptotic cell death. Cell.

[68] Nunez G, Merino R, Simonian PL, Grillot DA (1996). Regulation of lymphoid apoptosis by Bcl-2 and Bcl-XL. Adv Exp Med Biol.

[69] Choi KS, Kundu JK, Chun KS, Na HK, Surh YJ (2014). Rutin inhibits UVB radiation-induced expression of COX-2 and iNOS in hairless mouse skin: p38 MAP kinase and JNK as potential targets. Arch Biochem Biophys.

[70] Lee UE, Friedman SL (2011). Mechanisms of hepatic fibrogenesis. Best Pract Res Clin Gastroenterol.

[71] Hirota H, Yoshida K, Kishimoto T, Taga T (1995). Continuous activation of gp130, a signal-transducing receptor component for interleukin 6-related cytokines, causes myocardial hypertrophy in mice. Proc Natl Acad Sci U S A.

[72] Fischer P, Hilfiker-Kleiner D (2007). Survival pathways in hypertrophy and heart failure: the gp130-STAT3 axis. Basic Res Cardiol.

[73] Li PC, Chiu YW, Lin YM, Day CH, Hwang GY, Pai P (2012). Herbal Supplement Ameliorates Cardiac Hypertrophy in Rats with CCl(4)-Induced Liver Cirrhosis. Evidence-based complementary and alternative medicine: eCAM.

[74] Liu Y, Liu H, Meyer C, Li J, Nadalin S, Konigsrainer A (2013). Transforming growth factor-beta (TGF-beta)-mediated connective tissue growth factor (CTGF) expression in hepatic stellate cells requires Stat3 signaling activation. J Biol Chem.

[75] Wang X, Huang XJ, Ihsan A, Liu ZY, Huang LL, Zhang HH (2011). Metabolites and JAK/STAT pathway were involved in the liver and spleen damage in male Wistar rats fed with mequindox. Toxicology.

[76] Gao B (2005). Cytokines, STATs and liver disease. Cell Mol Immunol.

[77] Sansone P, Bromberg J (2012). Targeting the interleukin-6/Jak/stat pathway in human malignancies. Journal nkt Oncolo.

[78] Natarajan A, Wagner B, Sibilia M (2007). The EGF receptor is required for efficient liver regeneration. Proc Natl Acad Sci U S A.

[79] Suenaga M, Yamada S, Fujii T, Fuchs BC, Okumura N, Kanda M (2013). A functional polymorphism in the epidermal growth factor gene predicts hepatocellular carcinoma risk in Japanese hepatitis C patients. OncoTargets and therapy.

[80] Tanabe KK, Lemoine A, Finkelstein DM, Kawasaki H, Fujii T, Chung RT (2008). Epidermal growth factor gene functional polymorphism and the risk of hepatocellular carcinoma in patients with cirrhosis. Jama.

[81] Lin S, Saxena NK, Ding X, Stein LL, Anania FA (2006). Leptin increases tissue inhibitor of metalloproteinase I (TIMP-1) gene expression by a specificity protein 1/signal transducer and activator of transcription 3 mechanism. Mol Endocrinol.

[82] Jeong WI, Do SH, Jeong DH, Hong IH, Park JK, Ran KM (2006). Kinetics of MMP-1 and MMP-3 produced by mast cells and macrophages in liver fibrogenesis of rat. Anticancer Res.

[83] Fujii T, Fuchs BC, Yamada S, Lauwers GY, Kulu Y, Goodwin JM (2010). Mouse model of carbon tetrachloride induced liver fibrosis: Histopathological changes and expression of CD133 and epidermal growth factor. BMC Gastroenterol.

[84] Kuriyama S, Yokoyama F, Inoue H, Takano J, Ogawa M, Kita Y (2007). Sequential assessment of the intrahepatic expression of epidermal growth factor and transforming growth factor-beta1 in hepatofibrogenesis of a rat cirrhosis model. Int J Mol Med.

[85] Wang H, Lafdil F, Wang L, Yin S, Feng D, Gao B (2011). Tissue inhibitor of metalloproteinase 1 (TIMP-1) deficiency exacerbates carbon tetrachloride-induced liver injury and fibrosis in mice: involvement of hepatocyte STAT3 in TIMP-1 production. Cell & bioscience.

[86] Choi S, Lim TG, Hwang MK, Kim YA, Kim J, Kang NJ (2013). Rutin inhibits B[a]PDE-induced cyclooxygenase-2 expression by targeting EGFR kinase activity. Biochem Pharmacol.

